# Implications of SGLT Inhibition on Redox Signalling in Atrial Fibrillation

**DOI:** 10.3390/ijms22115937

**Published:** 2021-05-31

**Authors:** David Bode, Lukas Semmler, Christian U. Oeing, Alessio Alogna, Gabriele G. Schiattarella, Burkert M. Pieske, Frank R. Heinzel, Felix Hohendanner

**Affiliations:** 1 Center for Cardiovascular Research (CCR), Department of Internal Medicine and Cardiology, Campus Virchow-Klinikum, Charité University Medicine, Augustenburgerplatz 1, 13353 Berlin, Germany; lukas.semmler@charite.de (L.S.); christian.oeing@charite.de (C.U.O.); alessio.alogna@charite.de (A.A.); gabriele.schiattarella@charite.de (G.G.S.); burkert.pieske@charite.de (B.M.P.); Frank.heinzel@charite.de (F.R.H.); felix.hohendanner@charite.de (F.H.); 2 German Center for Cardiovascular Research (DZHK), Partner Site Berlin, 13353 Berlin, Germany; 3 Berlin Institute of Health (BIH), 13353 Berlin, Germany; 4 Translational Approaches in Heart Failure, Max Delbrück Center for Molecular Medicine in the Helmholtz Association, Robert Rössle Strasse 10, 13125 Berlin, Germany; 5 Department of Internal Medicine and Cardiology, German Heart Center Berlin, 13353 Berlin, Germany; 6 Department of Cardiology, Guy’s and St Thomas’ NHS Foundation Trust, London SE1 7EH, UK

**Keywords:** atrial fibrillation, heart failure, SGLT inhibition, cardiomyocytes, redox signalling, reactive oxygen species, oxidative stress, mitochondrial function, Ca^2+^ homeostasis

## Abstract

Atrial fibrillation (AF) is the most common sustained (atrial) arrhythmia, a considerable global health burden and often associated with heart failure. Perturbations of redox signalling in cardiomyocytes provide a cellular substrate for the manifestation and maintenance of atrial arrhythmias. Several clinical trials have shown that treatment with sodium-glucose linked transporter inhibitors (SGLTi) improves mortality and hospitalisation in heart failure patients independent of the presence of diabetes. Post hoc analysis of the DECLARE-TIMI 58 trial showed a 19% reduction in AF in patients with diabetes mellitus (hazard ratio, 0.81 (95% confidence interval: 0.68–0.95), n = 17.160) upon treatment with SGLTi, regardless of pre-existing AF or heart failure and independent from blood pressure or renal function. Accordingly, ongoing experimental work suggests that SGLTi not only positively impact heart failure but also counteract cellular ROS production in cardiomyocytes, thereby potentially altering atrial remodelling and reducing AF burden. In this article, we review recent studies investigating the effect of SGLTi on cellular processes closely interlinked with redox balance and their potential effects on the onset and progression of AF. Despite promising insight into SGLTi effect on Ca^2+^ cycling, Na^+^ balance, inflammatory and fibrotic signalling, mitochondrial function and energy balance and their potential effect on AF, the data are not yet conclusive and the importance of individual pathways for human AF remains to be established. Lastly, an overview of clinical studies investigating SGLTi in the context of AF is provided.

## 1. Introduction

Atrial fibrillation (AF) is the most common sustained atrial arrhythmia and a considerable global health burden. The prevalence of AF is rising, with currently up to 2% of the European population affected and a life-time risk of up to 1/3 individuals [1,2]. AF is associated with an increased risk for stroke (~5-fold), thrombo-embolism, reduced exercise capacity, hospitalisations for heart failure, left ventricular diastolic dysfunction and death [3,4,5,6]. AF is a multifactorial arrhythmia intertwined with other cardiovascular disorders, such as myocardial infarction, coronary artery disease, arterial hypertension, sleep apnea and lung disease [7]. Many of these disease entities share common risk factors (e.g., diabetes, obesity, advanced age). Currently, cornerstones in the treatment of AF are anticoagulation and rate or rhythm control therapies [8]. The superiority of either therapy regimen in the context of specific patient cohorts is still challenged and debated [9,10,11]. Other important therapeutic targets are underlying cardiovascular diseases, comorbidities and, if modifiable, risk factors [12]. Diabetes and AF commonly coexist: diabetes confers an odds ratio of 1.4 for men and 1.6 for women to develop AF [13]. Among diabetic patients, the prevalence of AF has been reported as ~15% [14]. Improved glycemic control prior to pulmonary vein isolation in diabetic patients has been linked to a strong reduction in AF recurrence [15]. However, data regarding the benefit of glycemic control on new-onset AF remains scarce.

Sodium-glucose-transporter inhibitors (SGLTi) are an antidiabetic drug class, developed to block SGLT in the distal (type 1) and proximal tubule (type 2) of the kidney glomerulus, thereby inhibiting glucose reabsorption of the kidney glomerulus. Different SGLTi show varying selectivity for type 2 over type 1 (e.g., Empagliflozin: 2680:1, Dapagliflozin 1242:1, Sotagliflozin 20:1). DAPA-HF and EMPEROR-Reduced evaluated the effect of SGLTi Dapagliflozin and Empagliflozin in 4.744 and 3.730 patients with heart failure with reduced ejection fraction (HFrEF), respectively. Interestingly, in both trials SGLTi exerted a beneficial effect on cardiovascular outcome, regardless of the presence or absence of diabetes [16,17]. The DECLARE-TIMI-58 trial examined cardiovascular outcomes of patients with type 2 diabetes upon Dapagliflozin treatment (n = 17.160). While not a primary outcome, post hoc analysis provided evidence for AF prevention unrelated to blood pressure or renal function [18]. These findings raise the intriguing question, whether SGLTi can prevent AF on-set and/or manifestation independently of glycemic control.

AF mechanistically arises from the combination of ectopic electric triggers and a vulnerable substrate maintaining abnormal electric activity [19,20]. Ectopic activity most commonly originates in the pulmonary veins [8,21]. Catheter-based isolation of pulmonary veins in patients with paroxysmal AF prevents new episodes at one-year follow-up in more than 75% of the cases [22]. Long-term reoccurrence following pulmonary vein isolation is most commonly facilitated by reconnection of a previously isolated region [8,22]. On the cardiomyocyte level, early and late after depolarisations are the result of a disturbed cellular ion homeostasis (Ca^2+^, Na^+^) and are the main mechanism of ectopic activity in pulmonary veins [21,23]. Structural remodelling of atrial myocardium (i.e., expansion of the extracellular matrix, cardiomyocyte apoptosis, rarefication of blood vessels and reduction in cell-cell contacts) provide a vulnerable substrate that facilitates re-entry and automaticity to maintain AF [19,24,25]. 

Perturbations in redox signalling are observed in a range of cardiovascular diseases including AF [26,27,28]. In general, redox signalling describes cellular signalling pathways sensitive to the oxidation-reduction balance of the cell, which is mainly determined by the presence of reactive oxygen species (ROS) [26]. These evolutionary conserved signalling networks fulfil a plethora of tasks in different tissues. In the heart, redox signalling regulates many physiologic processes, such as cell differentiation, hypertrophic growth and contractility [26]. However, hyperactive or uncoupled ROS signalling is a common driver of pathologic cardiac processes, such as fibrosis, hypertrophy and arrhythmia [29,30,31]. Concurrent involvement of ROS in physio- and pathologic signalling provide a potential explanation for inconsistent results seen with nonspecific ROS scavengers in cardiac disease [32]. In the context of AF, four primary mechanisms of enhanced ROS production are generally involved: mitochondria, xanthine oxidase, nicotinamide adenine dinucleotide phosphate-dependent oxidase (NOX) and uncoupled nitric oxide-synthases (NOS) [33]. Their individual significance is still an intense area of research [30,34,35]. Interestingly, a study in sheep suggested differential contribution of ROS sites during disease progression in AF. In their study, Reilly et al. initially observed enhanced NOX activity in early AF, while mitochondrial and uncoupled-NOS-related ROS release were predominant in disease advancement [35]. 

Accumulating evidence suggests that SGLTi counteracts cellular ROS production in cardiomyocytes, thereby potentially ameliorating AF burden [36,37,38,39]. Multiple mechanisms potentially linking SGLTi, redox signalling and AF have been described. Here, we will systematically review the current literature on cellular and systemic mechanisms linking SGLTi, redox signalling and the onset and progression of AF and discuss the evidence supporting their importance in human AF. Thereby, we will provide an up-to-date summary of the current knowledge and outline areas with demand for further research. Considering the immense healthcare burden AF represents, careful assessment of involved mechanisms will help to identify suitable patients with respect to co-morbidities, increase our understanding of redox signalling in AF, and will lay the groundwork for the development of new, targeted pharmacotherapies.

## 2. Mechanisms Linking ROS and AF

### 2.1. ROS, ECC and Cellular Na^+^ Balance

Disruptions of cardiomyocyte electrophysiology and its associated pathways have long been demonstrated to facilitate initiation, maintenance and progression of AF [20,23,40,41,42]. This section will focus on alterations of cellular ion homeostasis and excitation-contraction-coupling (ECC) which are subject to ROS signalling and SGLTi. More detailed descriptions of cellular electrophysiology in AF can be found elsewhere [20].

#### 2.1.1. Oxidation of Ca^2+^ Handling Proteins

ROS signalling tightly regulates ECC of cardiomyocytes via direct modification of Ca^2+^ handling proteins (e.g., sarco/endplasmatic retriculum Ca^2+^-ATPase (SERCA), ryanodine receptor (RyR)) and upstream protein kinases (e.g., Ca^2+^/calmodulin-dependent protein kinase II (CaMKII)) [43]. The RyR depicts the endpoint of Ca^2+^-induced-Ca^2+^-release (CICR) by releasing Ca^2+^ ions from the SR, thereby constituting one of the main integrators of cellular calcium homeostasis. An increased oxidation of the RyR complex has been observed in patients with chronic AF, which mediates an increased open probability of the channel [30,44]. In a physiological setting, stretch-dependent ROS release by NOX2 and consecutive RyR activation has been implied in the rapid inotropic response to mechanical force in cardiomyocytes [43]. However, exceeding or permanent activation of this pathway, as may be observed in conditions of pressure overload, renders cardiomyocytes vulnerable to leakage of Ca^2+^ ions from the SR to the cytosol [45,46]. Compensatory Na^+^/Ca^2+^ exchanger (NCX)-dependent extrusion of Ca^2+^ is accompanied by a positive net charge shift (1 Ca^2+^ outwards, 3 Na^+^ inwards), which has been directly associated with a higher DAD frequency in patients with persistent AF [47]. 

Following CICR, SERCA pumps Ca^2+^ ions from the cytosol to the SR, fulfilling two major functions integral to cellular ECC: (1) enabling cell relaxation by lowering cytosolic [Ca^2+^] and (2) restoring SR Ca^2+^ load for subsequent contraction. SERCA is reversibly deactivated in the presence of phospholamban (PLN; see *CaMKII* activity). ROS may alter SERCA activity depending on the type of ROS: oxygen-derived ROS have been demonstrated to depress SERCA function, possibly through direct protein oxidation of thiol groups [48,49]. However, S-glutathiolation by peroxinitrite, a nitric oxide-derived reactive molecule, has been shown to increase SERCA activity [49]. It remains unclear to what extent altered SERCA function is relevant to cellular arrhythmogenesis in AF. SERCA mediated Ca^2+^ uptake into the SR partially determines SR Ca^2+^ load. Conditions of increased SR Ca^2+^ load (SERCA ↑), as well as delayed Ca^2+^ reuptake (SERCA ↓), have been associated with an increased RyR leak [23,42,50].

#### 2.1.2. CaMKII Activity

Calcium and calmodulin-dependent protein kinase II (CaMKII), a key regulator of cellular Ca^2+^ homeostasis, has been demonstrated to affect function, expression and activity of ion channels in cardiomyocytes (e.g., RyR [51,52], L-type Ca^2+^ channel [53,54] and SERCA [55,56]). CaMK are a large group of enzymes with the ability to transfer phosphates from adenosine-triphosphate (ATP) to serine/threonine residues of other proteins, thereby regulating their structure and function [57]. The role of CaMKII in different settings of cardiovascular disease has been reviewed in-detail elsewhere [58,59,60]. An increased protein expression and activity of CaMKII has been observed in patients with chronic AF [47,61,62]. CaMKII is stimulated by several conditions linked to AF, such as hyperglycaemia, oxidative stress and beta-adrenergic activation [63,64,65]. Previous studies indicate that CaMKII increases open-probability of the RyR by phosphorylation, triggering an SR leak associated arrhythmic cascade (as described above). CaMKII phosphorylates PLN at Thr^17^, thereby relieving its inhibitory effect on the SERCA pump [66,67]. CaMKII oxidation is required in type 1 and 2 diabetic mice to develop enhanced AF susceptibility [68]. Myocardium-restricted transgenic overexpression of methionine sulfoxide reductase A, an enzyme reducing CaMKII oxidation, effectively ameliorated susceptibility for AF in angiotensin-2 treated mice, suggesting CaMKII oxidation as a viable therapeutic target in AF [64].

#### 2.1.3. SGLTi and Ca^2+^ Handling

Multiple studies have described a beneficial effect of SGLTi on cellular Ca^2+^ handling in cardiomyocytes of cardiometabolic disease models. Lee et al. studied ventricular cardiomyocytes of streptozotocin-induced diabetic rats after two weeks of oral Empagliflozin treatment [69]. Empagliflozin stimulated an initially depressed SERCA function, ameliorated RyR-mediated Ca^2+^ leak and restored SR Ca^2+^ load. Similar effects (accelerated Ca^2+^ removal, restored SR Ca^2+^ load) were observed in a rat model of carbohydrate-induced metabolic syndrome after 2 weeks of Dapagliflozin treatment [38]. In left atrial cardiomyocytes, we have been able to observe a reduction in cytosolic [Ca^2+^] and release amplitude of spontaneous Ca^2+^ release events in a rat model of metabolic heart failure with preserved ejection fraction (HFpEF) [70]. Interestingly, in murine and human cardiomyocytes, 24 h incubation with Empagliflozin in vitro reduced CaMKII activity [71]. Accordingly, RyR was less phosphorylated at sites that promote Ca^2+^ leak and the frequency of spontaneous Ca^2+^ release reduced. CaMKII stimulation provides a plausible upstream target for the beneficial effect on Ca^2+^ handling of SGLTi. 

#### 2.1.4. Na^+^ Balance

In addition to dysregulation of cellular Ca^2+^, alternations of Na^+^ currents in response to oxidative signalling were studied intensively. There is evidence of decreased peak Na^+^ current, increased late Na^+^ current, decreased Na^+^-K^+^-pump and increased Na^+^/H^+^ exchanger activity [40,41,72,73]. The elevated late Na^+^ current elongates action potential duration and renders cardiomyocytes vulnerable to early after depolarisations (EAD) [41]. Interestingly, Akar et al. observed decreased [Na^+^] in atrial myocardium after applying rapid pacing protocols in dogs [73], while studies in heart failure have repeatedly reported elevated [Na^+^] in the ventricular myocardium [74]. Disturbances of cytosolic [Na^+^] have been directly linked to mitochondrial formation of ROS in failing cardiomyocytes [75].

#### 2.1.5. SGLTi and Na^+^ Balance

The effect of SGLTi on intracellular Na^+^ balance in cardiomyocytes is matter of debate [76,77]. Studies in animal models suggest a decrease in cytosolic [Na^+^] after treatment with Empagliflozin, to which inhibition of the cardiac Na^+^/H^+^ exchanger and SGLT-1/2 have been proposed as central mechanisms [70,76,78,79]. However, conflicting evidence on the expression of SGLT subtypes 1 and 2 in the heart are present, which may be subject to species difference. Investigation of human heart tissue from healthy controls and different cardiac diseases found expression of SGLT 1, but not of SGLT 2 [80,81]. Conversely, we and others have observed expression of SGLT 2 in rat hearts [37,70]. Inhibition of the Na^+^/H^+^ exchanger and subsequent decrease in cellular [Na^+^] has been described in different animal models [76,78]. A recent study in human atrial cardiomyocytes reported Na^+^/H^+^ exchanger inhibition by Empagliflozin [82]. However, experiments in rat ventricular cardiomyocytes did not find an effect of SGLTi on Na^+^/H^+^ exchanger activity [77]. A cascade where SGLTi lower [Na^+^] and foster NCX dependent Ca^2+^ efflux have been suggested [70]. However, there is demand for further research given the contradictory reports on Na^+^/H^+^ exchanger activity and Na^+^ balance.

### 2.2. Mitochondrial Function

The functional integration of mitochondria into contractility and energy metabolism in the healthy and diseased heart involves a plethora of signalling pathways that have been recently reviewed [33,83,84]. In this section, we will focus on the interplay of cellular ion homeostasis, mitochondrial ROS release, energy production and the pathophysiology of AF. 

#### 2.2.1. Mitochondrial Ca^2+^-Regulated ROS Release

Mitochondrial [Ca^2+^] couples cellular ECC to mitochondrial ATP generation [85,86]. Mitochondrial Ca^2+^ influx is largely mediated by the voltage-dependent anion channel (VDAC) and the mitochondrial Ca^2+^ uniporter complex (MCU). Although the biophysical properties of the MCU have been studied extensively, the molecular composition has only recently been identified [87,88]. Mitochondria possess two lipid bilayer membranes. Ca^2+^ across the outer mitochondrial membrane is mainly facilitated by VDAC, while the MCU is responsible for Ca^2+^ influx into the mitochondrial matrix [89,90,91,92]. The contribution of other proteins (i.e., mitochondrial NCX) is still debated [93,94]. Cytosolic-mitochondrial [Ca^2+^] gradient and the inner mitochondrial membrane potential drive MCU-mediated Ca^2+^ influx [93]. In cardiomyocytes, Ca^2+^ efflux from the mitochondrial matrix mainly involves mitochondrial Na-Ca-Exchanger (mNCX)-mediated extrusion [95]. Mitochondrial matrix [Ca^2+^] itself stimulates key enzymes of the Krebs cycle (i.e., pyruvate-, α-ketoglutarate- and isocitrate dehydrogenase) and elevates the availability of NADH and FADH_2_ [96,97]. Increased substrate availability may then boost oxidative phosphorylation and regeneration of ATP via ligation of adenosine diphosphate (ADP) and inorganic phosphate [98]. By regulating the Krebs cycle, mitochondrial Ca^2+^ homeostasis directly alters mitochondrial redox state, which is determined by the ratio of nicotinamide adenine dinucleotide (NAD)H and flavin adenine dinucleotide (FAD)H_2_ to NAD^+^ and FAD [99]. The mitochondrial redox state and the generation of ROS are closely intertwined: Aon et al. demonstrated that deviation in both directions—towards an oxidised or reduced state—increases ROS release in cardiomyocytes [100]. Manipulation of mitochondrial Ca^2+^ content (i.e., by increasing cytosolic [Na^+^] and subsequent mNCX stimulation) is sufficient to increase mitochondrial redox-sensitive ROS release in cardiomyocytes [75]. Xie et al. have demonstrated an increased susceptibility to pacing-induced AF related to mitochondrial ROS release in mice harbouring a leaky RyR mutation [30]. Treatment with S107, a pharmacologic stabiliser of the closed state of RyR, attenuated ROS generation and AF susceptibility. 

#### 2.2.2. SGLTi and Mitochondrial ROS Release

There are several studies showing improved mitochondrial function and reduced ROS production in cardiomyocytes upon acute and chronic SGLTi. It appears plausible that extensive alterations in cytosolic Ca^2+^ cycling and Na^+^ balance benefit mitochondria in cardiomyocytes, but evidence for this link with regard to SGLTi has not been reported yet. Olgar et al. observed restoration of initially depleted mitochondrial [Ca^2+^] in ventricular cardiomyocytes of aged rats after acute treatment with Dapagliflozin in vitro [37]. Two-week oral treatment with Dapagliflozin in a rat model of metabolic syndrome improved mitochondrial function in ventricular myocardium as evident by increased inner mitochondrial membrane potential, restored ADP/ATP ratio and reduced levels of ROS [38]. Shao et al. demonstrated mitigation of left atrial enlargement, fibrosis and AF inducibility in diabetic rats following 8 weeks treatment with Empagliflozin [36]. Again, mitochondrial benefit manifested as increased maximum mitochondrial oxygen consumption, increased inner mitochondrial membrane potential and increased expression of proteins of mitochondrial biogenesis. Following 6 weeks treatment of Sotagliflozin in a rat model of HFpEF, left atrial cardiomyocytes exhibited decreased ROS production in a glucose-fasted state. This observation was accompanied by increased mitochondrial Ca^2+^ influx and improved mitochondrial swelling in response to Ca^2+^ [70]. 

### 2.3. Energy Balance and AMPK Activity

#### 2.3.1. Energetic Disturbances in AF

Cardiomyocytes have a very high demand for ATP production, turning over their ATP pool approximately every 10 seconds. As mitochondria supply > 95% of ATP, disturbances in mitochondrial metabolism constitute a powerful cellular stressor [101]. Decreased mitochondrial ATP production capacity has been observed in numerous human and animal models of AF [102,103]. Reviewing mechanisms facilitating mitochondrial disturbance of ATP production is beyond the scope of this review and the interested reader is referred to other literature [33]. It is noteworthy that increased cellular ROS release itself hampers mitochondrial function, thereby impairing ATP production and increasing ROS release in a feed-forward-mechanism [33,104]. 

Cardiomyocytes undergo metabolic adaptation to compensate for the decreased capacity of mitochondrial oxidation (despite an increased energy demand due to high beating frequencies in AF). This includes a shift of preferred energy substrates from fatty acids towards a more glycolysis-based metabolism [105,106], which has been indicated as a mechanistic driver of cellular arrhythmogenesis in cardiomyocytes: Zima et al. demonstrated that pharmacological depletion of mitochondrial ATP production was accompanied by increased glycolysis resulting in intracellular acidification (elevated lactate), increased [Na^+^] (increased Na^+^/H^+^-exchanger activity), higher diastolic [Ca^2+^] (increased NCX reverse-mode activity) and occurrence of delayed afterdepolarisations (DAD) [107].

#### 2.3.2. SGLTi and Myocardial Energy Utilisation

Chronic SGLTi treatment has been shown to elevate cardiac ATP availability and reduce rates of glycolysis in diabetic mice and in conditions of lipopolysaccharide-driven inflammation [108,109]. While these findings are noteworthy, the mechanisms of alternative fuel utilisation remain to be explored. Langendorff perfusion of Empagliflozin has been reported to decrease lactate generation in a Na^+^/H^+^-exchanger-dependent way and increase alpha-ketoglutarate synthesis from palmitate in diabetic mice [110]. We and others have shown that chronic treatment with SGLTi increases blood levels of ketone bodies, another potential substrate for cardiomyocyte energy metabolism [70,111]. Metabolomic and proteomic studies identified increased ketone bodies and key enzymes of ketone body metabolism in persistent AF [112]. However, mechanistic insights into ketone body metabolism and AF are scarce. In murine cardiomyocytes, 3-hydroxy-butyrate treatment induced acute inhibition of the transient K^+^ outward channel thereby delaying repolarisation, a potential inductor of DADs [113]. In contrast, ketone bodies have been proposed to mitigate inflammation and ameliorate adverse myocardial remodelling (see section on *ROS, inflammation and fibrosis)* [111]. 

#### 2.3.3. AMPK and AF

Adenosine monophosphate (AMP) activated protein kinase (AMPK), a serin/threonine kinase, is expressed in a multitude of tissues and regulates cellular energy usage and storage [114]. During cellular energy depletion, AMP and ADP activate AMPK allosterically [114,115]. Additional phosphorylation by upstream kinases (e.g., liver kinase B1 (LKB1) and CaMK) further stimulates AMPK activity and leads to a more than 1000-fold increase in activity [116]. Dephosphorylation by protein phosphatases (e.g., protein phosphatase 2a, protein phosphatase 2C) inhibits AMPK activity [117,118]. AMPK crucially regulates cellular metabolism, fostering katabolic fuel production and limiting energy consumption [119,120].

Cardiomyocyte-specific genetic knockout of AMPK activator LKB1 in mice causes the onset of paroxysmal AF that progresses into persistent AF [121,122]. LKB1 knockout mice thereby represent one of few rodent animal models that depict spontaneous AF [122]. Mice present atrial enlargement, infiltration with proinflammatory cells, downregulation of connexins and fibrosis at 8 weeks of age. Harada et al. have reported an increased AMPK activity in dogs following 1 week of rapid atrial pacing [119]. The same group also investigated AMPK expression and phosphorylation in human right atrial appendages of patients in sinus rhythm, paroxysmal AF and chronic AF (10 vs. 7 vs. 9 patients). The ratio of phosphorylated AMPK to total AMPK was found to be increased in paroxysmal AF but reduced in chronic AF [119]. The authors interpreted these results as a change of the AMPK activity during progression of AF.

Generally, AMPK favours a katabolic metabolism and hampers processes with high energy consumption. AMPK thereby inhibits crucial adverse remodelling pathways which are known to contribute to AF, such as fibrosis, hypertrophy and electrical remodelling [121,122,123]. Oral administration of acetylsalicylic acid and metformin, both AMPK activators, mitigates aforementioned remodelling processes and delays the occurrence of AF in LKB1 knockout mice [123]. In vitro, AMPK regulates alterations of intracellular Ca^2+^ homeostasis in response to metabolic stress [119,124]. In addition, AMPK functions as a central regulator of mitochondrial biogenesis, mitophagy and ROS release [120]. Mitochondrial ROS release has been shown to phosphorylate AMPK in cardiomyocytes, in turn limiting mitochondrial ROS release in a peroxisome proliferator-activated receptor gamma coactivator 1-alpha dependent way feedback inhibition loop [120]. These studies suggest AMPK activation as a powerful pharmacological target to alter mitochondrial ROS release in AF.

#### 2.3.4. SGLTi and AMPK

AMPK activation by SGLTi has been demonstrated in multiple tissues including hepatocytes and endothelial cells [125,126]. In murine cardiomyocytes, Empagliflozin has been reported to increase the amount of phosphorylated AMPK [127]. In accordance with this observation, oral treatment with Dapagliflozin (8 weeks) increased the ratio of myocardial phosphorylated to total AMPK [128]. In addition, incubation with Dapagliflozin for 16 h increases the ratio of phosphorylated to total AMPK in murine cardiac fibroblasts after exposure to lipopolysaccharides [128]. While these data appear promising, further investigations regarding SGLTi and AMPK activity in human myocardium are needed to verify these findings.

### 2.4. Inflammation and Fibrosis

#### 2.4.1. Inflammation in AF

Inflammation has been linked to the onset and progression of AF in a variety of animal models as well as in human disease. As a reflection of this, biomarkers of inflammation correlate with the onset of AF in humans and anti-inflammatory treatment has repeatedly been shown to convey a clinical benefit in terms of AF prevention (e.g., in postoperative settings) [129,130]. In a metanalysis by Salih et al., the authors show a significant reduction in postoperative AF upon anti-inflammatory treatment with colchicine with a number needed to treat as low as 7 in 1257 patients from 6 randomised controlled trial [131]. 

Among the drivers of inflammation in AF systemic medical conditions such as obesity, diabetes or hypertension constitute a very important group linking AF to other cardiac disease. AF itself was shown to promote inflammation thereby launching a vicious cycle that was suggested to contribute to the progression of AF [132]. Redox signalling pathways are an integral part of inflammatory processes. Please see Karam et al. and Pashkow et al. for a detailed review on the role of oxidative stress in heart disease [133,134].

Increased levels of ROS lead to the release of proinflammatory cytokines, the expression of adhesion molecules on endothelial cell to foster immune cell migration or the differentiation of naïve immune into mature immune cells [26,129]. Simultaneously, inflammation promotes the formation of ROS in immune cells and in cardiomyocytes [130,135]. Chronic inflammation has been associated with the development of AF through its role in atrial remodelling via several additional mechanisms: in murine pulmonary vein cardiomyocytes, tumour necrosis factor alpha (TNFα)—a proinflammatory cytokine—impairs Ca^2+^ handling and increases arrhythmogenic potential [136,137]. Additionally, inflammation impairs the conductance of electric signals between individual cells [25]. Proteins of the gap junction alpha family, commonly known as connexins, physiologically connect cardiomyocytes’ cytoplasm, thereby allowing propagation of the depolarisation from cell to cell [25]. Several inflammatory mediators downregulate the expression of connexins thereby slowing atrial electrical propagation and facilitating arrhythmogenicity [129]. Lastly, inflammation promotes atrial fibrosis. Transforming growth factor β (TGFβ), which is released in response to activation of TNFα receptor, activates cardiac fibroblasts. Activated fibroblasts impair Ca^2+^ handling of atrial cardiomyocytes through the release of paracrine mediators [138]. In addition, an orchestrated atrial extracellular matrix remodelling is triggered via the increase in myofibroblast proliferation, synthesis of collagen proteins and release of matrix metallopeptidases [24]. The resulting increase in non-cardiomyocyte fraction of the atria leads to heterogeneity in electrical conductance thereby generating a vulnerable substrate for AF [24]. 

#### 2.4.2. SGLTi and Systemic Inflammation

The effects of SGLTi on systemic inflammation and fibrosis are a topic of intense research. Preliminary studies in humans undergoing SGLTi treatment revealed changes in serum biomarkers of inflammation. Levels of the proinflammatory molecules leptin, c-reactive protein and TNFα were reduced in several studies [139]. These have been accompanied by an increase in adiponectin, a protein secreted by adipocytes that exhibits anti-inflammatory properties [139]. Additionally, direct anti-inflammatory effects on the myocardium have been reported. In vitro experiments with Empagliflozin in human and murine myocardium revealed suppression of makers of inflammation (TNF-α, IL-6, vascular cell adhesion molecule 1) and concomitant decrease in ROS signalling [140]. 

#### 2.4.3. SGLTi and Obesity

Obesity is a major risk factor for the development of AF [141]. Among the mechanisms underlying this association, induction of a proinflammatory state seems to play a very important role [141]. SGLTi mitigate the disadvantageous effects of obesity. Firstly, treatment with SGLTi reduces body weight and body fat mass in humans [142]. Secondly, studies on SGLTi in animals suggest a shift from brown towards white fatty tissue, that is less active in secreting inflammatory mediators [143]. Thirdly, epicardial fat volume is reduced in humans treated with SGLTi [144,145]. Importantly, epicardial fat highly correlates with the occurrences of cardiac fibrosis and has been shown to contribute via the secretion of proinflammatory cytokines [146]. 

#### 2.4.4. SGLTi and Myocardial Fibrosis

Besides its effects on obesity and fatty tissue and their proinflammatory properties, SGLTi also directly interacts with profibrotic pathways. Treatment with SGLTi in hypertensive heart disease, diabetic mice and human fibroblasts showed decreased profibrotic behaviour, collagen synthesis and myofibroblast activation [147,148,149]. Several mechanisms contribute to this: (1) Incubation with Empagliflozin reduced TGFβ-dependent activation of human myocardial fibroblasts and mitigates associated remodelling of the extracellular matrix [149]. (2) In animal models of obesity and diabetes, treatment with SGLTi shifts the polarisation of macrophages from the inflammatory M1 type to the more anti-inflammatory M2 type [150,151]. (3) In mice and human macrophages, SGLTi decrease the activity of the NLR family pyrin domain containing 3 inflammasome—a cellular component mainly found in macrophages, that facilitates maturation and secretion of the proinflammatory cytokines interleukin (IL) 1β and IL18, associated with myofibroblast activation [128,152]. Interestingly, this effect was found to be independent of the SGLTi effect on SGLT itself [128]. Possible mechanisms include the activation of the AMPK which inhibits inflammasome activation [128]. Additionally, increased plasma ketone body levels and decreased insulin levels decrease inflammasome activity in human macrophages [152]. The impact of SGLTi on fibrosis and AF was recently demonstrated in a canine rapid pacing model of AF [153]. Dogs that received continuous rapid pacing for three weeks were randomised to either placebo or Canagliflozin treatment. In the treatment group, ROS levels and fibrosis, and most importantly the inducibility of AF, were significantly reduced. These results are in agreement with previous studies in diabetic rats, where Empagliflozin significantly reduced atrial fibrosis and AF inducibility [36]. 

### 2.5. Conclusion on Potential Mechanisms Linking SGLTi, Redox Signalling and AF 

**Figure 1:**
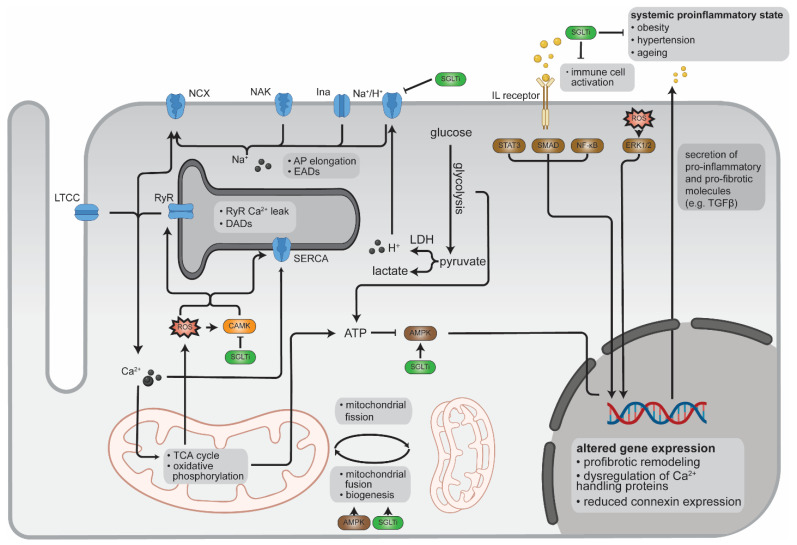
Proposed molecular mechanisms of SGLTi on redox signalling in cardiomyocytes.

The body of research concerning SGLTi and its role in cardiac disease is growing fast (results found in MEDLINE via Pubmed using the search term: “Sodium-Glucose Transporter 2 Inhibitors” [Mesh]) AND “Heart Diseases” [Mesh]: 2015: 8; 2020: 140). Accordingly, mechanisms that could explain for benefits seen with SGLTi in AF have been proposed as reviewed above (Figure 1). However, most studies are based on in vitro experiments or animal models. Only a little mechanistic evidence stems directly from experiments involving human samples (e.g., SGLTi effect on CAMKII activity in human cardiomyocytes, effects of SGLTi on systemic inflammation and obesity in humans, expression and phosphorylation of AMPK in human atrial samples). Therefore, the importance of the individual pathways in human AF and their potential to treat human AF remains to be demonstrated. 

## 3. Clinical Studies Investigating SGLTi in the Context of AF 

Even though several human landmark trials have shown a survival benefit and fewer hospitalisations for heart failure patients upon SGLTi treatment (DAPA-HF, EMPA-REG, CANVAS, DECLARE, EMPEROR-Reduced), human data on AF prevention are sparse. Granger et al. point out a small reduction in newly reported AF during the aforementioned trials even though the rate of strokes, potentially conferred through the occurrence of AF, were inconsistently altered [154].

Only recently, and in support of the notion that SGLTi reduces AF, an analysis of the DECLARE-TIMI 58 trial including 17,160 patients provided evidence for AF prevention in the setting of diabetes: the authors show a reduction in AF of 19% (hazard ratio, 0.81 (95% CI, 0.68–0.95)) upon treatment with dapagliflozin [18]. Interestingly, this reduction was independent of the presence of pre-existing AF or heart failure and was also not related to blood pressure or renal function.

Clinically, an important proposed mechanism for improved outcomes is an increased energy supply to the remodelled heart due to the increased plasma ketone levels that in turn increase cardiac ketone oxidation rates and reduces oxidative stress. Oxidative stress and advanced glycosylation endproducts have been associated with AF recurrence in patients undergoing pulmonary vein isolation [155]—a mitigation of oxidative stress through SGLTi might therefore contribute to a reduction in AF burden. Reliable prevention of AF would represent another pharmacological game-changer in the treatment of heart failure and its associated comorbidities. The effect on AF prevention by SGLTi remains to be tested in randomised controlled clinical trials.

## Abbreviations

AF	atrial fibrillation
AMP	adenosine monophosphate
ADP	adenosine diphosphate
AMPK	AMP activated protein kinase
ATP	adenosine triphosphate
CaMKII	Ca^2+^/calmodulin-dependent protein kinase II
CICR	Ca^2+^-induced-Ca^2+^-release
DAD	delayed afterdepolarisation
EAD	early afterdepolarisation
ECC	excitation-contraction coupling
FAD	nicotinamide adenine dinucleotide
HFpEF	heart failure with preserved ejection fraction
IL	interleukin
LKB1	liver kinase B1
MCU	mitochondrial Ca^2+^ uniporter
mNCX	mitochondrial Na^+^/Ca^2+^-Exchanger
NAD	nicotinamide adenine dinucleotide
NOX	nicotinamide adenine dinucleotide phosphate dependent oxidase
NOS	nitric oxide synthase
NCX	Na^+^/Ca^2+^ exchanger
PLN	phospholamban
ROS	reactive oxygen species
SERCA	sarco/endoplasmic reticulum Ca^2+^-ATPase
SGLTi	N^+^/glucose transporter inhibitors
SR	sarcoplasmic reticulum
RyR	ryanodine receptor
TNFα	tumor necrosis factor alpha
TGFβ	transforming growth factor β
